# The influence of depressive symptoms and substance use on adherence to antiretroviral therapy. A cross-sectional prevalence study: A influência dos sintomas depressivos e do uso de substâncias na adesão à terapia antirretroviral. Um estudo transversal de prevalência

**DOI:** 10.1590/1516-3180.2013.7450010

**Published:** 2014-09-19

**Authors:** Claudia Siqueira Tufano, Ricardo Abrantes do Amaral, Luciana Roberta Donola Cardoso, André Malbergier

**Affiliations:** I Medical student. Universidade Anhembi Morumbi, São Paulo, Brazil.; II MD, MSc, PhD. Lecturer at Medical School, Universidade de São Paulo (USP), São Paulo, Brazil.; III BSc, MSc. Postgraduate student at Medical School, Universidade de São Paulo (USP), São Paulo, Brazil.; IV MD, MSc, PhD. Lecturer at Medical School, Universidade de São Paulo (USP), São Paulo, Brazil.

**Keywords:** HIV, Depression, Medication adherence, Antiretroviral therapy, highly active, Immune system, HIV, Depressão, Adesão à medicação, Terapia antirretroviral de alta atividade, Sistema imunológico

## Abstract

**CONTEXT AND OBJECTIVE:**

Adherence to antiretroviral treatment (ART) is not a stable condition, but is dynamic, like mental conditions. The aim of this study was to examine whether non-adherence to ART is related to demographic and immunological variables, substance use and presence of depressive symptoms.

**DESIGN AND SETTING:**

This was a cross-sectional prevalence study carried out at a public AIDS treatment center in the city of São Paulo, Brazil, between July 2006 and January 2007.

**METHODS:**

438 patients on regular ART schedules with recent laboratory tests answered a demographic questionnaire, questions about substance use, the Hamilton Depression Rating Scale (HDRS) and the Simplified Medication Adherence Questionnaire (SMAQ).

**RESULTS:**

The prevalence of non-adherence over the past three months (a pattern of treatment interruption) was 46.3%, and 27.2% also reported this in the past week (a pattern of missed doses). ART interruption was significantly related to older age, lower CD4+ cell count and homosexual/bisexual transmission. The pattern of missed doses was significantly related to younger age, higher HDRS scores and higher viral load of RNA HIV.

**CONCLUSION:**

ART interruption may reflect recall errors and changes to the Brazilian demographic characteristics of HIV infection. The missed doses may reflect lifestyle characteristics of younger individuals. Attendance for HIV-positive individuals, particularly younger patients, should involve interventions and counseling in relation to the presence of depressive symptoms.

## RESUMO

**CONTEXTO E OBJETIVO:**

Adesão ao tratamento antirretroviral (TARV) não é uma condição estável, mas dinâmica, como os transtornos mentais. O objetivo deste estudo foi verificar se a não adesão ao TARV relaciona-se às variáveis demográficas e imunológicas, ao uso de substâncias e à presença de sintomas depressivos.

**DESENHO E LOCAL:**

Este estudo transversal de prevalência foi realizado em um serviço público para tratamento de pessoas HIV-positivas na cidade de São Paulo, Brasil, entre julho 2006 e janeiro 2007.

**MÉTODOS:**

438 pacientes em regime regular de TARV, que tinham exames laboratoriais recentes, responderam a questionário demográfico, questões sobre uso de substâncias, Escala de Hamilton para Depressão (HAM-D) e Questionário Simplificado de Adesão à Medicação (QSAM).

**RESULTADOS:**

A prevalência de não adesão nos últimos três meses (um padrão de interrupção de tratamento) foi de 46,3% e de 27,2% na última semana (um padrão de perda de doses). A interrupção do TARV relacionou-se significativamente a maior idade, menor contagem de células CD4+ e contágio homo/bissexual, enquanto o padrão de perda de doses relacionou-se significativamente com idade mais jovem, pontuação mais alta na HAM-D e maior carga viral de RNA HIV.

**CONCLUSÃO:**

A interrupção do TARV pode refletir erros de memória e mudanças nas características demográficas brasileiras da infecção por HIV. A perda de doses pode refletir aspectos de estilo de vida de indivíduos mais jovens. O atendimento de indivíduos HIV-positivos, particularmente os mais jovens, deve envolver intervenções e aconselhamento em relação à presença de sintomas depressivos.

## INTRODUCTION

Highly active antiretroviral therapy (HAART) is crucial for slowing down clinical progression and increasing survival among HIV-infected individuals. However, to achieve this clinical effect, adherence to HAART is necessary.^1^^,^^2^ Conceptually, adherence is “the extent to which the patient’s behavior matches the prescriber’s agreed recommendations”.^3^

Among the multifactorial causes of non-adherence to antiretroviral therapy (ART),^4^ it has been observed that substance use^5^^,^^6^^,^^7^ and depressive symptoms^4^^,^^8^ have an impact. Substance use disorders and mood disorders are the most common psychiatric diseases among HIV-positive patients, and have been shown to affect people living with HIV (PLWH) significantly more often than the general population.^9^ Additionally, stressful events and trauma have a negative effect on important components of the complex immune system, such as CD4 T cells.^10^^,^^11^

Since adherence is unstable in situations such as mental conditions^12^ with erratic^13^ and dynamic courses,^14^^,^^15^ or is expressed in other forms, such as missed doses or as treatment interruptions, i.e. so-called “drug holidays”,^16^ early detection of non-adherence may prevent the development of ART resistance and treatment failure.^17^

In Brazil, few studies have investigated the impact of substance use or depressive symptoms on adherence to ART. Previous results showed that patients on ART may interrupt treatment to consume alcohol,^18^^,^^19^ or that using alcohol, tobacco and any illicit drug could be linked to a profile of higher vulnerability to non-adherence.^20^^,^^21^ The same vulnerability was observed for severe depressive symptoms^22^ or depression.^23^

## OBJECTIVE

The aim of this study was to examine whether non-adherence to ART over the past three months, also called treatment interruption (i.e. more than two days of interruption), and over the last week, also called missed doses, are associated with the same demographic and immunological variables and, particularly, with substance use over the past month and presence of depressive symptoms over the past week.

## METHODS

### Study population

This study was carried out by the Alcohol and Drug Study Group (GREA) of the psychiatric department of a medical school at a public university in the city of São Paulo, Brazil, and at the HIV treatment center (AIDS Clinic) affiliated to this medical school. Since 1994, this center has been providing multidisciplinary HIV/AIDS care on a 12-hour day outpatient basis. The center provides services relating to infectious diseases, gynecology, psychiatry, obstetrics, endoscopy, oral health, psychology, social work, nutrition and physical education. High-complexity patients are followed up in accordance with the Brazilian Ministry of Health protocols.^24^

The inclusion criteria for this study were that the patients needed to be over 18 years of age and on ART, and to have HIV-related laboratory data (CD4+ cell count and viral load) recorded within the last three months preceding the interview. The exclusion criteria were a clinical diagnosis of dementia or a score of less than 24 points on the Mini-Mental State Examination (MMSE).^25^

The study consisted of face-to-face interviews conducted by trained psychiatrists and psychologists. Every day, the first five patients scheduled for routine medical consultations were invited to participate in the study. All interviews were conducted in a private room and lasted approximately two hours. The study was carried out from July 2006 to January 2007.

A sample of 455 was obtained from the total population of patients under treatment (N = 3,000), taking 20% to be the estimated prevalence of non-adherence to ART, alpha = 5%, and a confidence interval (CI) of 95%.

#### Measurements criteria

Alcohol and drug use was assessed over the preceding month. Last-month alcohol use was measured as none/less than once a week or more than once a week.

The Hamilton Depression Rating Scale (HDRS)^26^ was used to measure the presence of depressive symptoms.

The Simplified Medication Adherence Questionnaire (SMAQ)^27^ was used to assess adherence to ART. SMAQ has a dichotomous structure and its questions can be assessed separately: (1) “Do you ever forget to take your medicine?”; (2) “Are you careless at times about taking your medicine?”; (3) “If at times you feel worse, do you stop taking your medicine?”; (4) “Thinking about the last week, how often have you not taken your medicine?”; (5) “Did you not take any of your medicine over the last weekend?”; (6) “Over the past three months, on how many days have you not taken any medicine at all?” The non-adherence was considered to be “positive” when a non-adherent patient was detected, i.e. when more than two doses were missed over the past week (Question 4) or over the last weekend (Question 5) (recent non-adherence, i.e. a pattern of missed doses), or more than two days of total non-medication during the past three months (Question 6) (non-adherence over the past three months, i.e. a pattern of treatment interruption).

### Clinical features

The most recent CD4+ cell count and RNA HIV viral load registered in the patient’s records were assessed*.* Plasma RNA viral load was described as log_10_ RNA copies/ml. Viral load was classified in categories as follows: detectable (HIV RNA copies > 40) or undetectable (HIV RNA copies ≤ 40).

### Statistical analysis

The following variables were defined as predictive: gender (male or female); race/ethnicity (declared by the interviewee as white, black, mixed or other); education was defined as basic (up to 11 years of study) or higher (college or university level); working (yes or no); conjugal situation (living together or living alone); the form of HIV transmission was described as heterosexual sexual intercourse, homosexual/bisexual sexual intercourse, hemodialysis/transfusion (named intravenous HIV transmission) or unknown transmission route; alcohol consumption last month (none/less than once a week or more than once a week); cocaine use (no or yes); and cannabis use (no and yes). Continuous variables were described as means and standard deviation (SD); age and time since HIV seroconversion were described in years; annual income was described in United States (US) dollars (US dollar to Real exchange in January 2007 = 2.133); HDRS score, number of CD4+ cells and viral load were described as above.

The outcome variable was adherence to ART, which was defined as adherence over the past three months (past adherence, treatment interruption; yes or no) and as adherence over the past week (recent adherence, missed doses; yes or no). Pearson’s correlation between the two measurements of adherence was not significant (*r* = - 0.002; P = 0.97). Categorical data were described as percentages, and Pearson’s chi-square test was used to make comparisons relating to past and recent adherence status. Student’s t test was used for continuous variables relating to the measurements of adherence. A probability level below 5% was considered statistically significant.

The magnitude of the correlation between predictive variables relating to past and recent ART non-adherence was measured by examining the crude odds ratio (OR) and the corresponding 95% confidence intervals (CI). The best predictive models for past and recent non-adherence were built through multiple logistic regression analyses using stepwise selection for each adherence measurement. Independent variables with significant crude OR results (P ≤ 0.1) were entered into the initial model. The data were analyzed using SPSS version 16.0 (SPSS Inc., Chicago, Illinois, USA).

### Ethical procedures

This study was approved by the university’s Ethics Committee. All subjects included in the study gave their informed consent prior to their inclusion. The project was supported by a grant (CSV 083/06) from the HIV/STD National Coordination Office of the Brazilian Ministry of Health.

## RESULTS

### Demographics and clinical and adherence status of the sample

Between July 2006 and January 2007, 459 patients on antiretroviral treatment were assessed. Twenty-one patients were excluded: three because of low MMSE scores (< 24 points) and eighteen because no CD4+ cell counts and HIV viral loads recorded over the last three months were available.

The 438 patients included in the study were predominantly white (n = 251; 57.3%) and living alone (n = 299; 68.3%). The mean age was 41.7 years (standard deviation, SD ± 8.0) and the gender distribution was approximately equal (males: n = 226; 51.6%). Close to 75% of the sample had basic education and close to 25% had higher education. Almost 62% of the sample (n = 240) were working, and the mean annual income of those who were currently working or receiving a pension was US$ 11,056 (SD: ± 10,428), ranging from US$ 1,283 to US$ 91,397. The mean length of time since becoming aware of being HIV infected was 9.46 years (SD: ± 4.7). The main form of HIV transmission was through heterosexual sexual intercourse (n = 241; 55%). Twenty-seven percent of the sample (n = 118) reported having become infected through homosexual/bisexual transmission, and 16% (n = 71) said that they did not know what their HIV transmission route was. The remaining 2% (n = 8) of the sample reported intravenous HIV transmission. No patient reported injection drug use.

The mean CD4 cell count was 569.1/ml (318.4). Mean log_10_ RNA copies/ml among the sample was 1.96 (SD: ± 1.2).

Over the preceding month, 364 patients (83.1%) reported no alcohol use or use less than once a week. Last-month alcohol use more than once a week was reported by 74 individuals (16.9%). Over the preceding month, almost 10% of the sample (n = 41) reported use of illicit drugs: 34 (7.8%) reported cannabis use, 16 (3.7%) cocaine use and 0.2% (n = 1) amphetamine use. Frequent recent use (more than once a week) of cannabis and cocaine were reported, respectively, by 5.1% (n = 22) and 1.4% (n = 6) of the sample. The mean score for the HDRS was 6.97 (SD: ± 8.3). Overall, 319 patients (72.8%) reported some pattern of non-adherence to ART. Three patients who reported past and recent non-adherence were included in the past non-adherent group (Table 1).

**Table 1.**
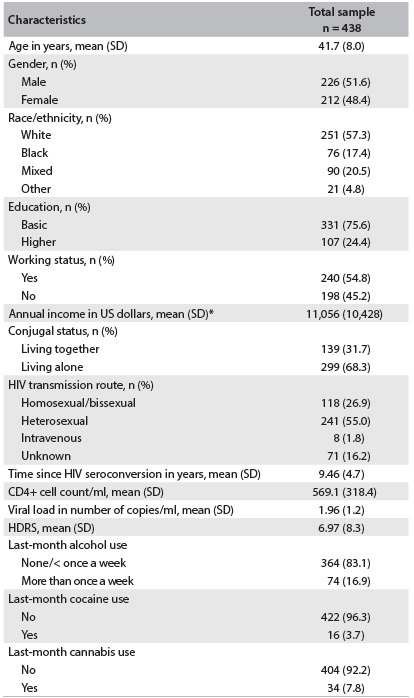
Total sample characteristics at the HIV treatment center, in 2007

SD = standard deviation; US = United States; HDRS = Hamilton Depression Rating Scale; HIV = human immunodeficiency virus. *Mean annual income of those who were currently working or receiving a pension.

### Past non-adherence

Two hundred and three patients (46.3%) on ART reported past non-adherence. The correlation between past adherence and undetectable viral load was not significant (Pearson chi-square value = 0.038; P = 0.845; OR = 1.04; 95% CI: 0.67-1.62). The mean age of past non-adherent patients was higher than that of the adherents (mean age 41.6 years). The likelihood of being non-adherent was almost 40% lower among higher-educated patients. In comparison with patients who reported homosexual/bisexual HIV transmission, those who reported heterosexual transmission or an unknown route had a lower likelihood of being non-adherent (more than 50% and 90%, respectively). Past non-adherent patients had lower CD4+ cell counts than adherents. Reports of last-month alcohol use more than once a week were marginally associated with past non-adherence to ART (OR = 1.04; 95% CI: 0.63-1.72).

### Last-week non-adherence

One hundred and sixteen patients (26.5%) reported having adhered to ART over the past week. Almost 80% of the adherent patients had undetectable viral loads (Pearson chi-square value = 7.89; P = 0.005; OR = 1.94; 95% CI: 1.21-3.09). Recent non-adherence was significantly associated with younger age and higher numbers of HIV RNA copies, in comparison with recent adherent patients. Patients who reported recent non-adherence also showed higher HDRS scores than the adherent patients. Last-month alcohol use more than once a week was also marginally correlated with recent non-adherence. All the data on adherence, past non-adherence and recent non-adherence is presented in Table 2.

**Table 2.**
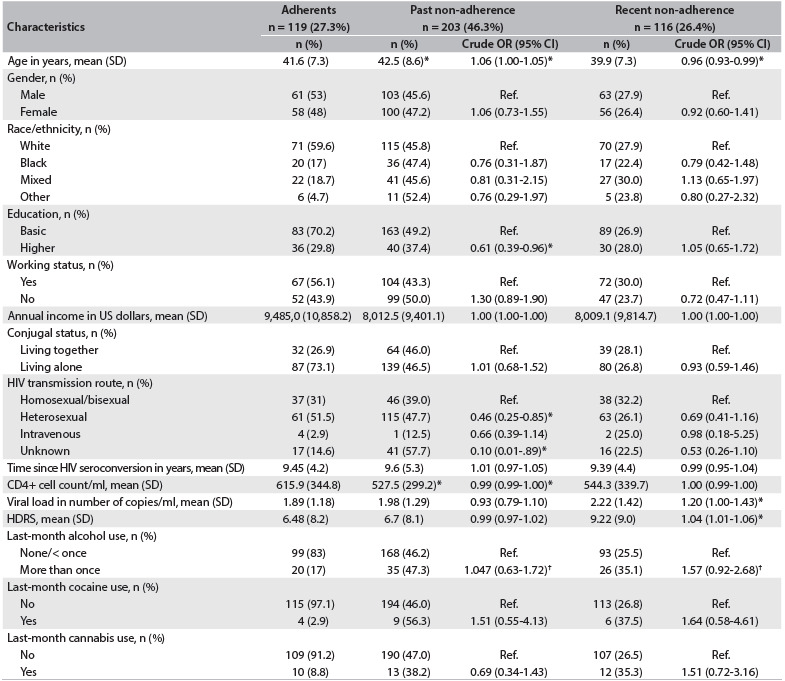
Bivariate associations between predictive variables and past and recent non-adherence to antiretroviral therapy at the HIV treatment center, in 2007

*P < 0.05, ^†^P < 0.01. OR = odds ratio; CI = confidence interval; SD = standard deviation; US = United States; HDRS = Hamilton Depression Rating Scale; HIV = human immunodeficiency virus; Ref. = 1.0.

### Multiple regression results

Past non-adherence to ART was independently predicted by older age and lower CD4+ cell count. Homosexual/bisexual transmission was also significantly and independently associated with non-adherence, in comparison with those who reported heterosexual and unknown transmission routes. For recent non-adherence to ART, the strongest independent predictors were younger age and higher HDRS scores. Recent non-adherence was also independently predicted by higher numbers of HIV RNA copies (Table 3).

**Table 3.**
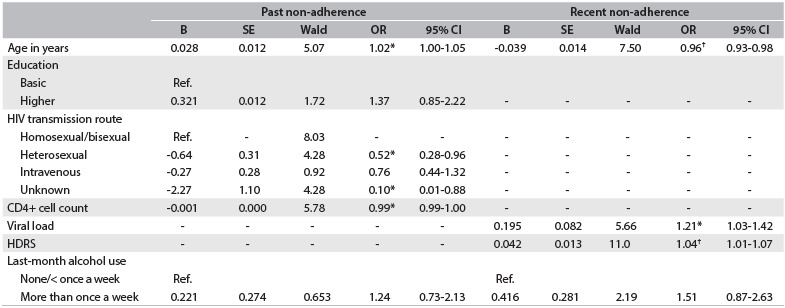
Multiple logistic regression results for predictable variables and past and recent non-adherence to ART among patients at the HIV treatment center in 2007

*P < 0.05, ^†^P < 0.01. OR = odds ratio; CI = confidence interval; HDRS = Hamilton Depression Rating Scale; HIV = human immunodeficiency virus. B = beta; SE = standard error; Wald = Wald test.

## DISCUSSION

In this sample of HIV individuals on ART, adherence was evaluated as two patterns: over the past three months and over the past week, which were defined as treatment interruption and missed doses, respectively. The rate of recent non-adherence (26.4%) was close to what was observed by Nemes et al. (25%) in 322 healthcare clinics in Brazil,^28^ but lower than what was observed in other Brazilian studies (36.9% and 37.2%),^21^^,^^22^ with different methodologies. In a randomized controlled trial, the percentages of non-adherence measured by means of Medication Event Monitoring System (MEMS) caps ranged from 35.4% to 50.9%,^29^ which was similar to the results for past non-adherence in the present study.

The frequency of past non-adherence was almost twice the recent frequency. Since past adherence covered a longer time interval, this result was not unexpected. Additionally, the past and recent non-adherence results came from different individuals, as shown by the correlation results, and it is probably correct to say that they came from different treatment behaviors.

Previous studies have shown that the higher the adherence is, the lower the likelihood of virological failure will be,^30^^,^^31^ as seen in the binary association, with almost twice the likelihood of having a detectable viral load among patients who missed doses. Since the viral load is a faster and more accurate method for signaling the clinical consequences of treatment failure^32^ than the CD4+ cell count, which may be related to other immunological variations,^33^ it is possible that a recent non-adherence pattern will also have the same ability, compared with past non-adherence.

More importantly, since adherence does not provide a full explanation for the observed variations in treatment responses,^34^ it was observed that the effect of non-adherence, as a consequence of missed doses, was related to growing levels of depressive symptoms. This correlation is well known, but it has been suggested that some methodological issues concerning the time of recall, measurements of depression and past and present history of depression act as misleading factors.^35^ In our study, HDRS was used to measure depression symptoms over the last week, and it is likely that measurements of both recent adherence and depressive symptoms would express a pattern of missed dose frequency, and would also be related to the detectable viral load.^36^

Along with the impact of depressive symptoms on the immune system, the influence of alcohol and other drug use over the past month was not related to recent non-adherence to ART. Last-month alcohol use was measured as none/less than once a week or as more than once a week. This last measurement admits a range of patterns of consumption that might be acting as a confounding factor. Nevertheless, previous studies have shown that adherence would be higher among alcohol users than among abstainers (or those who drank relatively less).^37^

Concerning younger age, some difficulties in taking medication at weekends because of stigma,^38^ or due to alterations in these individuals’ regular schedules or involvement in other activities, might lead to missing doses of ART. Additionally, the U-shape between age and depressive symptoms^39^ could have an exponential effect on adherence.

Past adherence or treatment interruption is more likely to be affected by recall errors than is recent adherence, which may be strengthened by the independent association of older age and unknown transmission route among non-adherent patients over this interval. However, multivariate analysis also showed the magnitude of the correlation of lower CD4+ count and heterosexual transmission with treatment interruption. Taking into account the changes in the demographics of HIV infection in Brazil, with the growth of heterosexual transmission and increasing life expectancy,^40^ it is likely that such changes require shifts in prevention and care.^41^

Because the period used to define past adherence comprised a three-month interval, the CD4+ cell count results might have expressed prior conditions, and even previous virological elevations. It is also possible that an age-related effect and the sparseness of preventive actions for heterosexual individuals might explain the joint effect of poor immune response.

Some limitations of the present study need to be discussed. Firstly, although self-reporting is affected in several ways by personal difficulties, it is generally credible^42^ and correlates with clinical data,^31^ as seen in our study. As recommended by Simoni et al.,^43^ we proposed item standardization for the SMAQ, through applying the last two questions, which cover recent adherence. This allowed the patients to distinguish between missing doses and treatment interruption.

Additionally, the sample studied was not randomly selected from all HIV patients in Brazil, since the patients were selected from a specific center. Nevertheless, this study sample was not statistically different from the center population. Even so, further generalization is limited to similar populations.

These results do not allow the extension of such findings to a clinical condition of major depression, which requires a full clinical evaluation, and presumably a specific treatment. Notwithstanding this, our results emphasize that clinicians should integrate the delivery of clinical care, targeted interventions^44^ and counseling^45^^,^^46^ against depressive symptoms through regular appointments, in order to avoid poorer outcomes. These findings should stimulate clinicians to evaluate depression when the patient is not complying with the treatment or is in a situation of treatment failure.

Future research should replicate these findings using a standardized clinical diagnosis of depression.

## CONCLUSION

Our results show that depressive symptoms were related to the presence of patterns of missing doses, non-adherence to treatment and virological failure, which should be targeted by clinicians through regular appointments.
